# Elastin degradation products in acute lung injury induced by gastric contents aspiration

**DOI:** 10.1186/s12931-018-0873-1

**Published:** 2018-08-31

**Authors:** Pedro Ayala, Raúl Vivar, Rebeca Montalva, Pablo Olmos, Manuel Meneses, Gisella R. Borzone

**Affiliations:** 1 Department of Respiratory Diseases and Medical Research Center, Pontificia Universidad Católica de Chile, Marcoleta 350, piso 1, Santiago, Chile; 2 Department of Diabetes and Nutrition, Pontificia Universidad Católica de Chile, Santiago, Chile; 3 Pathology Unit, Instituto Nacional del Tórax, Santiago, Chile

**Keywords:** Elastinolysis, Elastin degradation, Gastric fluid aspiration, Acute lung injury, Extracellular matrix

## Abstract

**Background:**

Gastric contents aspiration is a high-risk condition for acute lung injury (ALI). Consequences range from subclinical pneumonitis to respiratory failure, depending on the volume of aspirate. A large increment in inflammatory cells, an important source of elastase, potentially capable of damaging lung tissue, has been described in experimental models of aspiration. We hypothesized that in early stages of aspiration-induced ALI, there is proteolytic degradation of elastin, preceding collagen deposition. Our aim was to evaluate whether after a single orotracheal instillation of gastric fluid, there is evidence of elastin degradation.

**Methods:**

Anesthesized Sprague-Dawley rats received a single orotracheal instillation of gastric fluid and were euthanized 4, 12 and 24 h and at day 4 after instillation (*n* = 6/group). We used immunodetection of soluble elastin in lung tissue and BALF and correlated BALF levels of elastin degradation products with markers of ALI. We investigated possible factors involved in elastin degradation and evaluated whether a similar pattern of elastin degradation can be found in BALF samples of patients with interstitial lung diseases known to have aspirated. Non-parametric ANOVA (Kruskall-Wallis) and linear regression analysis were used.

**Results:**

We found evidence of early proteolytic degradation of lung elastin. Elastin degradation products are detected both in lung tissue and BALF in the first 24 h and are significantly reduced at day 4. They correlate significantly with ALI markers, particularly PMN cell count, are independent of acidity and have a similar molecular weight as those obtained using pancreatic elastase. Evaluation of BALF from patients revealed the presence of elastin degradation products not present in controls that are similar to those found in BALF of rats treated with gastric fluid.

**Conclusions:**

A single instillation of gastric fluid into the lungs induces early proteolytic degradation of elastin, in relation to the magnitude of alveolar-capillary barrier derangement. PMN-derived proteases released during ALI are mostly responsible for this damage. BALF from patients showed elastin degradation products similar to those found in rats treated with gastric fluid. Long-lasting effects on lung elastic properties could be expected under conditions of repeated instillations of gastric fluid in experimental animals or repeated aspiration events in humans.

## Background

Gastric contents aspiration is a high-risk condition for lung injury. Consequences range from subclinical pneumonitis to diffuse alveolar damage and progressive respiratory failure, depending on the volume of aspirate, with fibrosis development in some patients [1, 2].

Various experimental approaches have been used to gain insight into the pathogenesis and pathophysiology of aspiration-induced lung injury. Instillation of individual components of gastric fluid has contributed to the understanding of their relative roles in lung injury [3]. Whereas hydrochloric acid instillation results in derangement of the alveolar-capillary barrier with edema and an intense inflammatory reaction [4–14], instillation of acid-free gastric food particles induces a delayed inflammatory reaction, followed by granuloma formation without significant edema [15–17]. Synergistic effects have been reported when acid and gastric food particles are instilled in combination [4, 9]. Few studies have used the whole gastric fluid to study the pathogenesis of aspiration. Those studies have used small volumes of gastric fluid instilled into small areas of the lung with the aim of answering questions regarding lung transplant rejection [15–17].

Our group has addressed the study of the continuum of changes after a single event of bilateral aspiration of whole gastric contents and has shown that a single orotracheal instillation of gastric fluid in the rat lung results in severe acute lung injury with several histological similarities to diffuse alveolar damage (DAD), that evolves to an organization process involving intraluminal plugs of myofibroblasts and collagen fibers, affecting small bronchioles, alveolar ducts and peribronchiolar alveolar spaces, associated with particle-containing foreign-body giant cells either isolated or forming granulomas that later resolves [18]. This sequence of events reflects important remodeling of lung extracellular matrix (ECM) involving deposition and degradation of its components. Most studies regarding mechanisms involved in ECM remodeling after acute insults to the lung have focused on deposition of new ECM components, mainly collagen deposition [8, 15] but very few have evaluated ECM degradation [19–21]. Elastin, a polymer of tropoelastin is a major component of the lung ECM providing the lung with elasticity, tensile strength, and stability [22]. Increased catabolism of elastin can be detected by a reduction in mature elastin content or by the release of elastin-degradation products after mature elastin breakdown. In this regard, evidence for a reduction in mature elastin content has been unexpectedly obtained in fibrotic diseases such as usual interstitial pneumonia (UIP) and cryptogenic organizing pneumonia (COP) using modern non-invasive microscopy technology [23], whereas elastin-degradation products have been documented in animal models of acute lung injury ending in fibrosis [19, 20] and in human diseases as diverse as chronic obstructive pulmonary disease (COPD) [24], acute respiratory distress syndrome (ARDS) [25] and, idiopathic pulmonary fibrosis (IPF) [26]. Interestingly, all these disease conditions have also been associated with gastric contents aspiration [4, 27].

In our model, a 15- to 20-fold increase in bronchoalveolar lavage fluid (BALF) total cell count was found in the first 24 h after a single instillation of gastric fluid, with polymorphonuclear (PMN) cell predominance [18], an important source of elastase and free radicals, with the potential of damaging lung elastic tissue [28]. In addition to the inflammatory reaction induced by aspiration, elastic tissue damage could be produced by the direct effect of gastric fluid or could be part of the changes that take place in the remodeling of lung ECM after aspiration.

We hypothesized that in gastric fluid aspiration-induced ALI there is proteolytic degradation of elastin preceding collagen deposition.

Our aim was to evaluate at different time points during the course of ALI induced by a single instillation of gastric fluid whether there is evidence of elastin degradation in lung tissue and BALF. We used Western blot analysis to detect lung elastin degradation products and correlated the presence of these products in BALF and lung tissue with markers of acute lung injury. In addition, we studied if these degradation products are present in BALF samples of human patients with interstitial lung diseases (ILDs) that have evidence of aspiration.

We found that a single orotracheal instillation of gastric fluid into the rat lung is associated with early degradation of lung elastin. The significant positive correlation found with PMN cell count in BALF suggests that neutrophil elastase could be involved, since exogenous elastase produces a similar pattern of elastin degradation products. Evaluation of BALF from patients with ILD who have evidence of aspiration revealed the presence of elastin degradation products similar to those found in BALF of rats treated with gastric fluid.

These results are important to be considered, since repetitive aspirations of gastric contents could result in long-lasting alterations of lung elastic properties.

## Methods

The study was performed according to a protocol submitted to and approved by the Animal Research Ethics Committee of the Pontificia Universidad Católica de Chile in adult male Sprague-Dawley rats (270–300 g).

### Rat model of single orotracheal instillation of gastric fluid

#### Gastric contents pool

Adult male Sprague-Dawley rats fasted overnight were anesthetized i.p. with xylazine-ketamine (5.1 and 55.1 mg/kg, respectively) to obtain gastric fluid through a gastrotomy. Gastric fluid samples were pooled, filtered through a 100 um mesh, and kept at -80 °C. Animals were euthanized thereafter by exsanguination under anesthesia.

#### Orotracheal instillation of gastric fluid

Under the same anesthetic protocol, another set of animals was orotracheally intubated with a 22 gauge wire-fed catheter. A modified human otoscope (Welch Allyn, Skaneateles Falls, NY) was used to visualize the glottis. A volume of gastric fluid previously determined by the authors (data not shown) to distribute evenly (1.5 mL/kg, pH 1.69) was instilled, and animals were allowed to recover spontaneously from anesthesia.

#### Study groups

**Fig. 1:**
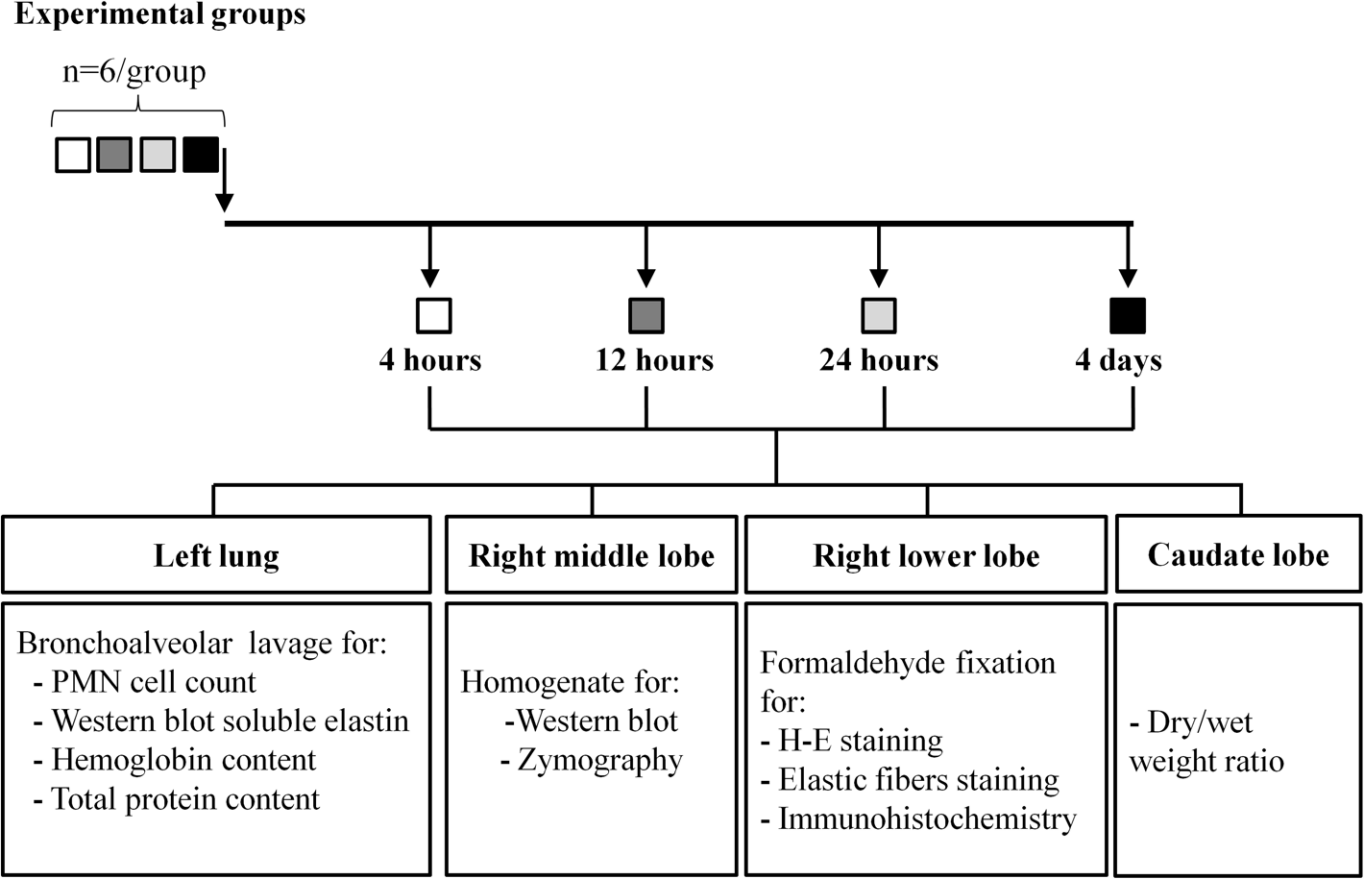
Diagram showing animal groups, timing of tissue sampling and analysis

Histological and biochemical studies were performed at 4, 12 and 24 h and at day 4 after instillation (*n* = 6 per group). Animals without intervention (*n* = 6) served as controls since they did not differ significantly from saline treated animals. Diagram in Fig. 1 shows animal groups, tissue sampling and analysis.

### Sample collection

Lungs were excised *en bloc*, and the left main bronchus was cannulated for bronchoalveolar lavage (BAL). For each animal, three aliquots of 0.15 M saline (1 mL each) were instilled, immediately aspirated and pooled. Total and differential cell count was obtained using a Neubauer chamber and a cytospin slide centrifuge (StatSpin Cytofuge 2; Iris, Westwood, MA). Cytoslides were stained with DiffQuik (QCA, Tarragona, Spain). After centrifugation, BALF was stored at -80 °C until used for the measurement of hemoglobin concentration, total protein content, and western blot analysis of soluble elastin. The right middle lobe was excised, frozen and later homogenized for western blot analysis of soluble elastin and for matrix metalloproteinase-9 (MMP-9) and − 2 (MMP-2) activities by zymography. The right lower lobe was fixed at 20 cm H_2_O with 10% buffered formaldehyde solution and paraffin embedded for histological studies. The caudate lobe was used to obtain the wet/dry weight ratio.

### Histologic evidence of tissue injury

For each animal, four right lower lobe longitudinal sections were embedded in paraffin, sectioned at 5 μm, and stained with hematoxylin-and-eosin. A board-certified pathologist (M.M.) scored samples according to ATS statement [29]. Scores for PMN cells in alveolar spaces, PMN cells in the interstitium, proteinaceous debris and alveolar septal thickening were used to correlate with soluble elastin. In addition, sections underwent specific staining of the elastic system using the Unna-Taenzer acid orcein stain [30].

### Western blot analysis of soluble elastin

Equal amounts of protein extracts from lung homogenates or BALF were heat-denatured in Laemmli sample buffer with 2-mercaptoethanol (5%), resolved in 10% SDS-PAGE gel and transferred to nitrocellulose membranes (Thermoscientific, Rockford, IL, USA). Next, blots were blocked with 5% PBS-nonfat dry milk for 1 h at room temperature and then incubated with a goat polyclonal anti-elastin primary detection antibody (1:1000) (sc-17,580 Santa Cruz Biotechnology, Dallas, Texas, USA) overnight at 4 °C. After thoroughly washing with PBS 0.05% Tween-20, membranes were incubated for 2 h at room temperature with a rabbit anti-goat HRP-conjugated secondary antibody (1:5000) (Thermo Scientific, Rockford, IL, USA). Elastin fragment immunoreactivity was visualized by enhanced chemiluminescence (SuperSignal™ Pico Chemiluminescent Substrate kit; Thermo Scientific, Rockford, IL, USA). C-DiGit Blot Scanner (Li-Cor, Lincoln, NE, USA) was used to image chemiluminescent signals by scanning. Densitometric analysis was performed using the ImageJ software version 1.46 m (NIH, Bethesda, MD). β-tubulin was used to control for equal loading.

Animal and human samples were studied using this method. In addition, samples from control rats treated “in vitro” with HCl (pH: 1.69, 37 °C, for 2,4,6 or 8 h) or pancreatic elastase (0.5 μM, pH:8 and 10 min incubation) were also studied.

### Acute lung injury markers

**Lung wet/dry weight ratio** of the caudate lobe was obtained using an oven at 60 °C until stable dry weight was achieved.

**Total protein concentration** in BALF was measured using the Bradford assay.

**Hemoglobin concentration** in BALF was measured by light absorbance at 510- to 650-nm wavelength using a spectrophotometer (Shimadzu, Kyoto, Japan).

**Lung tissue MMP-9 and MMP-2 activities:** Gelatinolytic activity of these lung tissue MMPs was studied using zymography [31]. Equal amount of lung tissue homogenate total protein (30 μg) were loaded into a gelatin-containing electrophoresis gel (10% polyacrylamide and 1% gelatin under non-reducing conditions). After electrophoresis, gels were washed in 2.5% TritonX-100 (Sigma-Aldrich, St. Louis, MO) to remove SDS, incubated overnight at 37 °C in a calcium containing developing buffer, stained with 0.1% Coomassie Brilliant Blue and destained until areas of gelatinolytic activity became evident. Densitometric analysis was performed using ImageJ software version 1.46 m (NIH, Bethesda, MD).

### Analysis of BALF samples from patients with exacerbation of interstitial lung diseases

BALF samples obtained from six  patients with an acute exacerbation of their ILD as part of their routine clinical evaluation were studied in a similar way as the rat samples. They all exhibited evidence of gastric contents aspiration, since they all had high levels of BALF pepsin. As controls for this part of the study, we used six BALF samples from patients without interstitial lung disease who required bronchoscopy for the study of a pulmonary nodule and had no evidence of aspiration, since they all had negative BALF pepsin levels.

### Statistical analysis

Non-parametric analysis of variance (Kruskall-Wallis) was used because of the small sample size. Linear regression analysis and Spearman’s rank correlation were also used [32]. Unless otherwise noted, the results are expressed as median values, interquartile range and range. A *p* value < 0.05 was considered statistically significant. Analyses were performed using GraphPad Prism 5.0 software.

## Results

### Histological evaluation of acute lung injury in the first 4 days after a single orotracheal instillation of gastric fluid

**Fig. 2:**
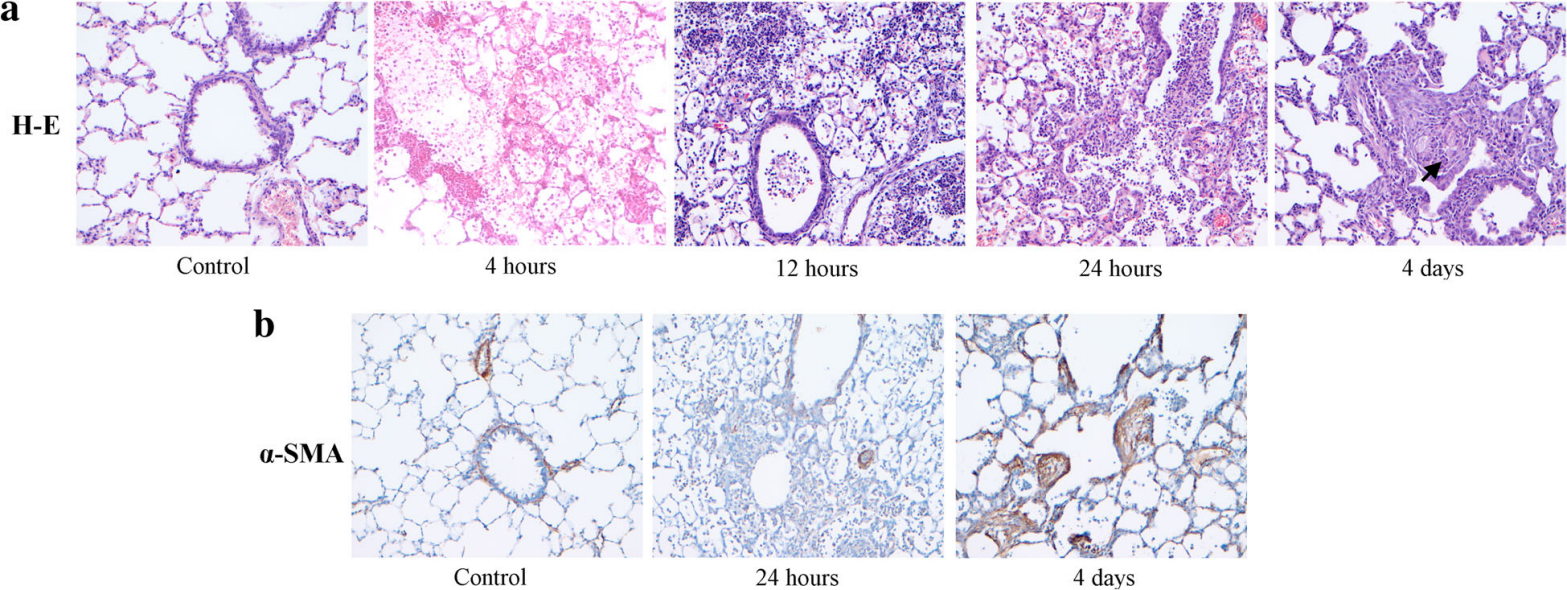
Histological evaluation of acute lung injury in the first 4 days after a single orotracheal instillation of gastric fluid. **a** Light microscopy (hematoxylin and eosin stain) of lung from a control animal and from animals studied 4, 12 and 24 h and at day 4, after gastric fluid instillation. Polymorphonuclear neutrophils and red blood cells with abundant intra-alveolar proteinaceous material are seen at 4 h. A more intense reaction is seen at 12 and 24 h. At day 4, markers of ALI, as seen in the first 24 h, are no longer observed. Instead, intra-alveolar buds of granulation tissue, characteristic of OP containing giant-cell granulomas are seen. Arrow: giant-cell granuloma inside a Masson body. Original magnification: 200X. **b** Light microscopy (alpha-SMA immunostaining) of control lung and lung of animals studied at 24 h and at day 4 after gastric fluid instillation. The control and 24-h samples exhibit alpha-SMA (brown) staining localized to the wall of bronchioles and blood vessels only. Intra-alveolar alpha-SMA-positive structures (myofibroblasts) are observed only at day 4. Original magnification: 200X

Histological changes in the first 4 days after instillation are shown in Fig. 2. Figure 2a shows the time course of changes with H-E staining. At 4 h there is increased alveolar thickening by interstitial edema and inflammatory cell infiltration, along with abundant protein-rich intra-alveolar exudate containing neutrophils and red blood cells, adopting a peri-bronchiolar distribution. These changes become more intense at 12 and 24 h, with patchy consolidation, due to coalescence of affected areas. At day 4, markers of ALI, as those described in the first 24 h are no longer observed. Instead, intra-alveolar buds of granulation tissue, characteristic of organizing pneumonia (OP) are seen, sometimes containing granulomas and giant cells.

Figure 2b shows alpha-SMA immunostaining of both, control lung and lung of animals studied at 24 h and at day 4 after gastric contents instillation. The control and 24-h samples exhibit alpha-SMA (brown) staining localized only to the wall of bronchioles and blood vessels, without intra-alveolar alpha-SMA-positive structures, which are only seen at day 4.

### Evidence of damage to the lung elastic fiber system in animals treated with a single instillation of gastric fluid and studied at 4, 12 and 24 h and at day 4 after instillation

**Fig. 3:**
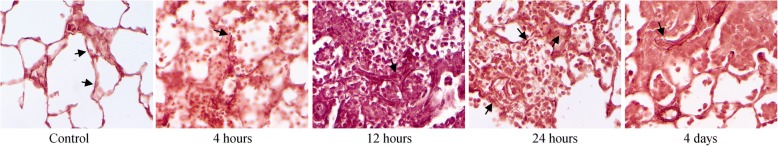
Evidence of damage to the lung elastic fiber system in animals treated with a single instillation of gastric fluid and studied at 4, 12 and 24 h and at day 4 after instillation. Representative fields illustrating elastic fiber system distribution in lung samples from control and acute lung injury induced by gastric fluid. Elastic fibers are stained in deep violet within alveolar walls (arrows). Photographs were taken at an original magnification of 600X from slides stained with orcein

Figure 3 shows the elastic fiber system distribution in lung samples from a control animal and from animals with acute lung injury induced by gastric fluid. The control sample shows preserved architectural pattern of the elastic system. Samples in the first 24 h after instillation show sparce and fragmented bundles of elastic system fibers.

At each of the time points studied, we observed elastic fiber fragmentation in areas with inflammatory reaction and not in preserved areas.

At day 4, with significantly less inflammatory cells, elastic fiber fragmentation was less evident and localized only to the alveolar septa adjacent to intra-alveolar fibrosis. Interestingly, elastic fiber fragmentation was not observed inside Masson bodies.

### BALF total and differential cell count in the first 4 days after a single orotracheal instillation of gastric fluid

Changes in total and differential cell count in BALF are shown in Table 1. A 15- to 20-fold increase in total cell count was seen in the first 24 h, with PMN cell predominance. By day 4, there was a return to mononuclear cell predominance.

**Table 1:** BALF total and differential cell count

	Total cell count (mean ± SD)	% PMN	% MN
Control	8.2 ± 3.1 × 10^4^	0.8	99.2
4 h	1.7 ± 0.9 × 10^6^	82	18
12 h	7.8 ± 3.9 × 10^5^	82	18
24 h	1.2 ± 0.5 × 10^6^	77	13
4d	1.5 ± 0.7 × 10^5^	12	88

*SD* standard deviation, *PMN* polymorphonuclear, *MN* mononuclear

### Soluble elastin in lung tissue homogenate and BALF after gastric fluid instillation

**Fig. 4:**
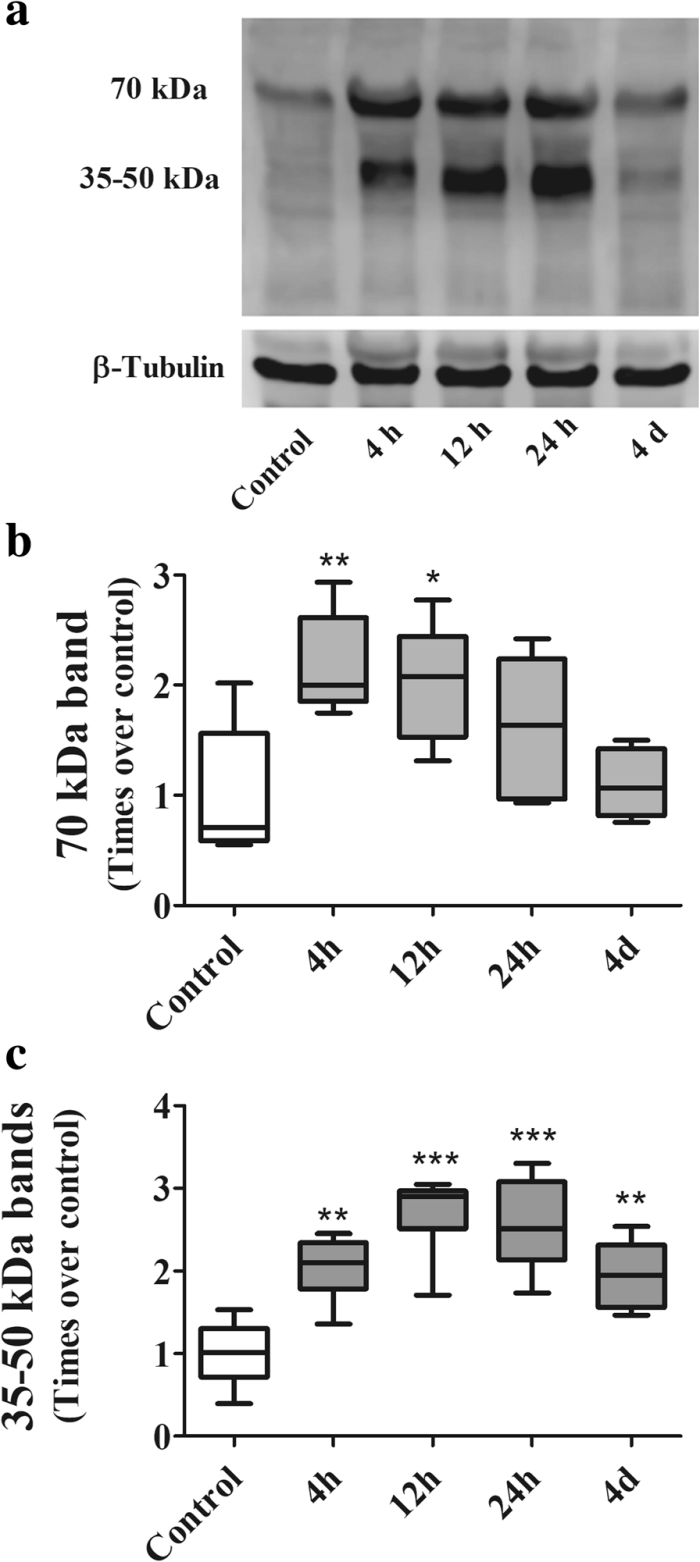
Soluble elastin in lung tissue homogenate of control animals and animals treated with a single instillation of gastric fluid and studied at 4, 12 and 24 h and at day 4 after instillation, as determined by Western blotting. **a** The immunoblot shows that the control sample exhibits a single band of soluble elastin with a molecular weight of 70 kDa, likely corresponding to tropoelastin. In samples from treated animals, this band is accompanied by small molecular weight bands in the 35–50 kDa range. The 70 kDa band density increases at 4 h and shows a slight progressive reduction thereafter. Band densities in the 35-50 kDa range are largely increased at 4 h, exhibit a further increase at 12 and 24 h and are significantly smaller at day 4. β-tubulin immunoblot shows equal protein loading. **b** Densitometric analysis of the 70 kDa Western blot band (*n* = 6) normalized to beta-tubulin and expressed as times over control. After a significant increase in band density at 4 h, a progressive reduction to reach control level at day 4 is seen. Data are presented as median values, interquartile range and range. **: *p* < 0.01; *: *p* < 0.05 with respect to controls and day 4. **c** Densitometric analysis of the 35–50 kDa Western blot bands corresponding to elastin-degradation fragments (*n* = 6) normalized to beta-tubulin and expressed as times over control. These band densities increase significantly at 4 h, remain elevated at 12 and 24 h and decrease without reaching control levels at day 4. Data are presented as median values, interquartile range and range. **: *p* < 0.01; ***:*p* < 0.001 with respect to controls. h: hours, d: days

Figure 4 shows the results of soluble elastin immunodetection in lung tissue homogenate. In Fig. 4a, the immunoblot of the control sample shows a 70 kDa band, likely corresponding to tropoelastin, whereas smaller molecular weight bands in the 35–50 kDa range corresponding to elastin degradation products are barely detectable. In treated animals, the 70 kDa band exhibits variable size and is accompanied by bands in the 35–50 kDa range, with variable densities depending on time after instillation. Figure 4b and c show the densitometric analysis of these bands. In Fig. 4b, the 70 kDa band at 4 h exhibits a peak increment in density of 2.2 times the control band (*p* < 0.01). Later on, this band decreases progressively to become similar to the control band at day 4 (r_S:_ − 0.6515; *p* < 0.01). In Fig. 4c, the 35–50 kDa bands show a progressive increment up to 24 h (rs: + 0.7376; *p* < 0.001) and although these bands decrease in size at day 4, they are still detectable.

**Fig. 5:**
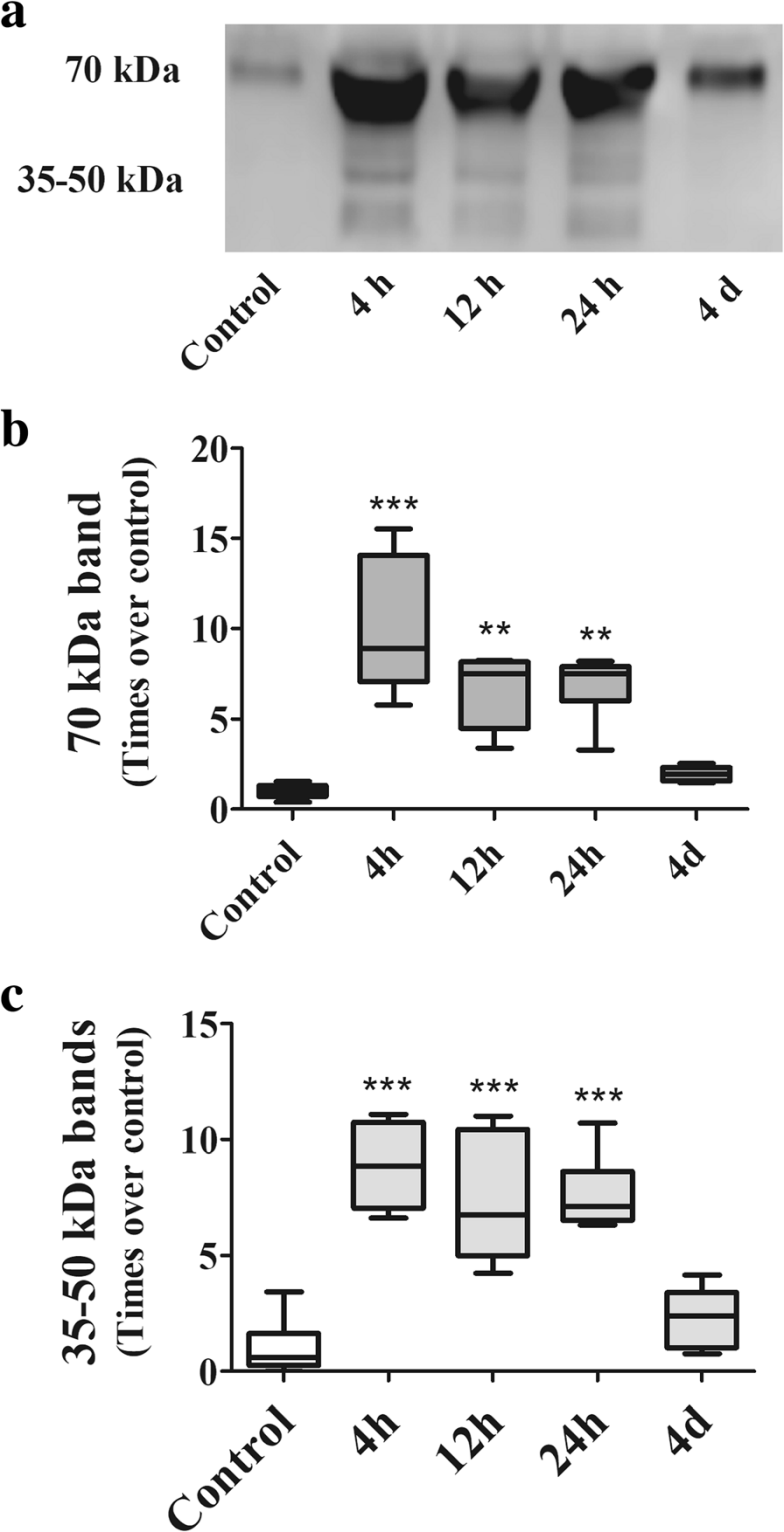
Soluble elastin in BALF from control animals and animals treated with a single instillation of gastric fluid and studied at 4, 12 and 24 h and at day 4 after instillation, as determined by Western blotting. **a** The immunoblot shows that the control sample exhibits only a 70 kDa band, likely corresponding to tropoelastin. This band is significantly enlarged at 4 h, remains enlarged at 12 and 24 h and returns to control level at day 4. The 35–50 kDa bands, not present in the control sample, are present only at 4, 12 and 24 h after instillation. **b** Densitometric analysis of the 70 kDa Western blot band (*n* = 6) expressed as times over control. After a significant increase in band density at 4 h, a progressive reduction to reach control level at day 4 is seen. Data are presented as median values, interquartile range and range. **: *p* < 0.01; ***: *p* < 0.001 with respect to controls and day 4. **c** Densitometric analysis of the 35–50 kDa Western blot bands corresponding to elastin-degradation fragments (*n* = 6) expressed as times over control. These band densities increase significantly at 4 h, remain elevated at 12 and 24 h and return to control levels at day 4. Data are presented as median values, interquartile range and range. ***: *p* < 0.001 with respect to controls and day 4. h: hours, d: days

Figure 5 shows the results of soluble elastin in BALF. In Fig. 5a, the immunoblot of the control sample shows a single band of soluble elastin with a molecular weight of 70 kDa. Bands in the 35–50 kDa range are non-detectable. As in lung tissue homogenate, in treated animals, the size of the 70 kDa band is variable depending on time after instillation and is accompanied by small molecular weight bands in the 35–50 kDa range. Figure 5b and c show the densitometric analysis of these bands. In Fig. 5b, the 70 kDa band at 4 h exhibits a peak increment in density of 10 times the control band (*p* < 0.001). Later on, it decreases progressively to become similar to the control band at day 4 (r_S:_ − 0.5599; *p* < 0.01). In Fig. 5c, band densities in the 35–50 kDa range are visible 4 h after instillation. Later on, these bands decrease progressively in density to become similar to the median density of the control samples at day 4 (r_S:_ − 0.6676; *p* < 0.001).

The time course of changes in band densities shows that changes in tropoelastin slightly precede changes in small molecular weight elastin-derived peptides, mainly in lung tissue homogenates. Whereas the peak increment for the 70 kDa band in lung tissue homogenates is observed at 4 h, the peak increment for the 35-50 kDa bands is seen between 12 and 24 h after instillation.

### In vitro effect of acid and exogenous elastase on the pattern of lung tissue elastin degradation

**Fig. 6:**
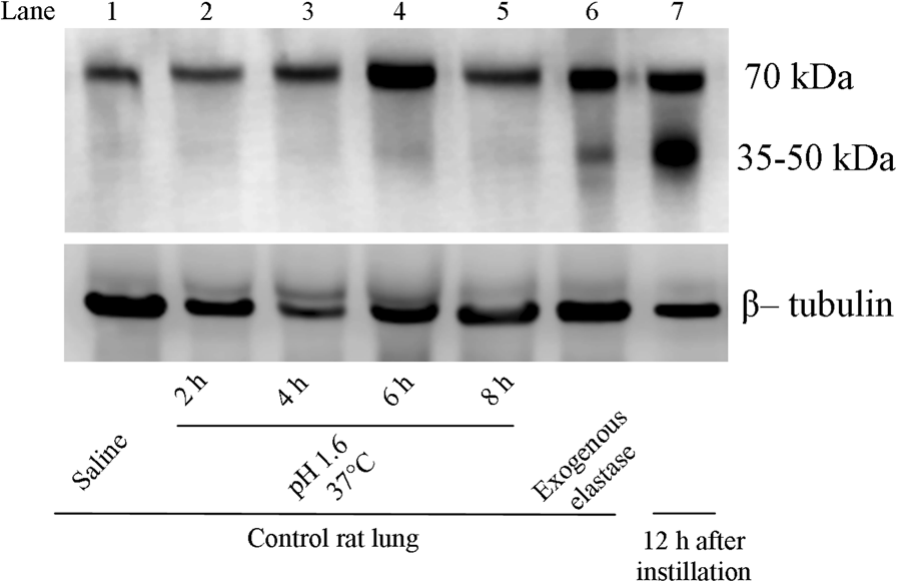
In vitro tropoelastin degradation pattern is not dependent on acidity but it is associated with the presence of elastase. Representative Western blot of soluble elastin illustrating the effect of acid and elastase on the pattern of elastin fragmentation. Lane 1 corresponds to lung tissue homogenate from a control rat showing only the 70 kDa band, likely corresponding to tropoelastin. Lanes 2 to 5 correspond to lung tissue homogenate from a control rat treated with hydrochloric acid at 37 °C to a final pH:1.6, for 2, 4, 6 and 8 h, showing the 70 kDa band, without evidence of smaller molecular weight elastin-derived peptides. Lane 6 corresponds to lung tissue homogenate from a control rat treated with commercially available porcine pancreatic elastase showing the 70 kDa band and a pattern of fragmentation similar to that seen in lane 7, corresponding to lung tissue homogenate from a rat studied 12 h after gastric fluid instillation. β-tubulin immunoblot shows protein loading. h: hours

Figure 6 shows a representative Western blot of soluble elastin illustrating the effects of acid and exogenous elastase on the pattern of elastin degradation in samples of control lung in vitro. Lane 1 corresponds to lung tissue homogenate from a control rat sample exposed to saline, showing only the 70 kDa band, likely corresponding to tropoelastin. Lanes 2 to 5 correspond to lung tissue homogenate from a control rat sample treated with hydrochloric acid at 37 °C to a final pH:1.6, for 2, 4, 6 and 8 h, showing only the 70 kDa band, without evidence of elastin degradation over time. Lane 6 corresponds to lung tissue homogenate from a control rat sample treated with porcine pancreatic elastase showing the 70 kDa band and a pattern of elastin degradation that is similar to that seen in lane 7, corresponding to lung tissue homogenate from a rat studied 12 h after gastric fluid instillation. The small molecular weight elastin-derived fragments (35–50 kDa) observed in this lane are similar to those obtained when using exogenous elastase in normal rat lung.

### Correlations between soluble elastin and markers of acute lung injury

**Fig. 7:**
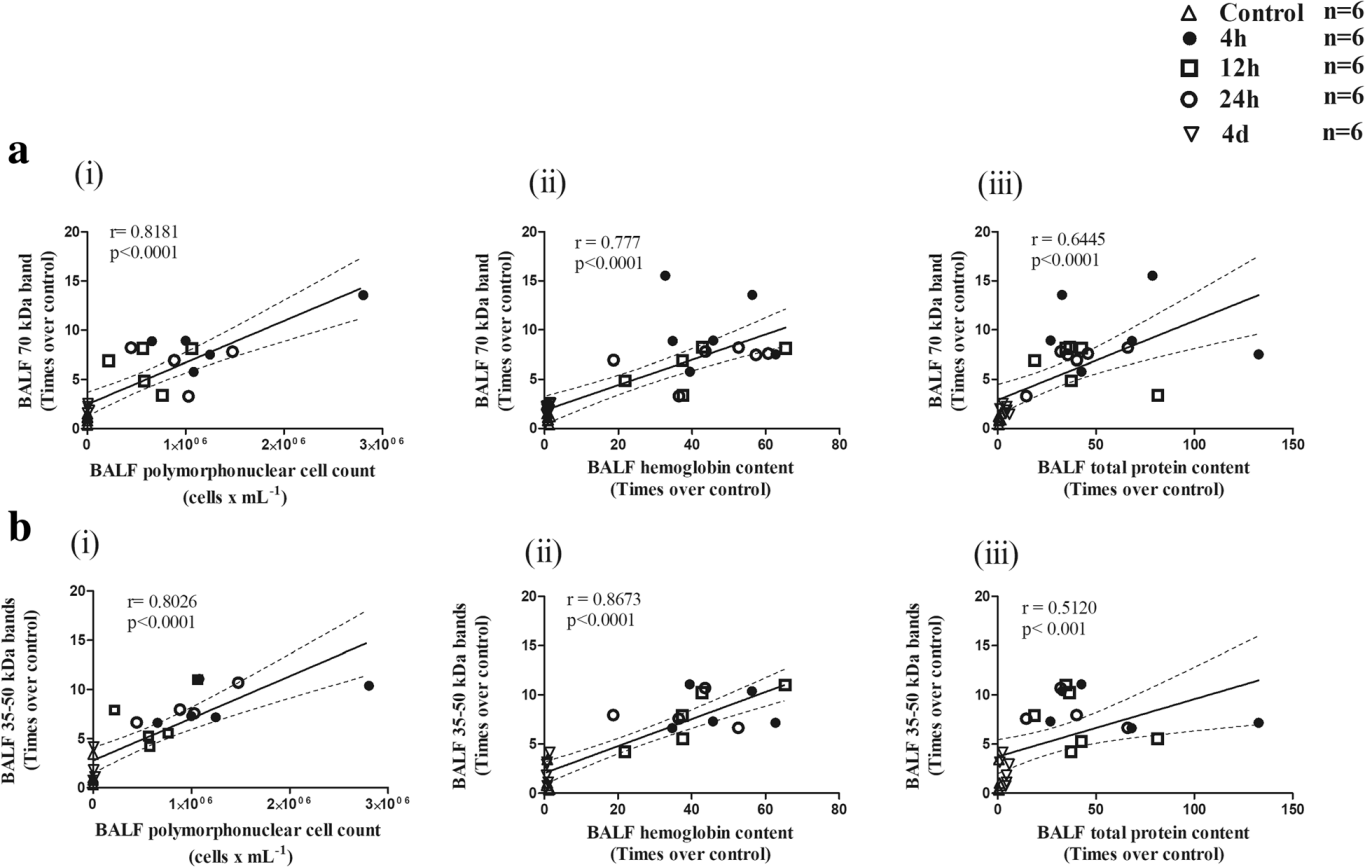
Correlations between soluble elastin and markers of acute lung injury in BALF**. Panel a: S**ignificant positive correlations between the 70 kDa band density found in BALF and: i) the PMN cell count, ii) the hemoglobin content and iii) the total protein content in BALF. **Panel b:** Significant positive correlations between the 35–50 kDa band densities found in BALF and: i) the PMN cell count, ii) the hemoglobin content and iii) the total protein content in BALF

Table 2 and Fig. 7 illustrate the correlations between soluble elastin as determined by Western blotting and several markers of ALI . Table 2 shows no correlation between any of the bands corresponding to soluble elastin and both the wet/dry weight ratio and MMP-9 activity. Only MMP-9 data was used for correlations since MMP-2 activity did not change in the study period. Figure 7a shows **s**ignificant positive correlations between the 70 kDa band density found in BALF and: a) the PMN cell count (*r* = 0.8181, *p* < 0.0001) b) the hemoglobin content (*r* = 0.777, *p* < 0.0001) and c) the total protein content (*r* = 0.6445, *p* < 0.0001) in BALF. Figure 7b shows significant positive correlations between the 35–50 kDa band densities found in BALF and: a) the PMN cell count (*r* = 0.8026, *p* < 0.0001), b) the hemoglobin content (*r* = 0.8673, *p* < 0.0001) and c) the total protein content (*r* = 0.5120, *p* < 0.001) in BALF. In addition, significant positive correlations were found between the 70 kDa band and several histological markers of acute lung injury and between the 35–50 kDa bands and the same histological markers (Table 2).

**Table 2:** Correlations between soluble elastin and markers of acute lung injury (ALI)

ALI marker	70 kDa band	35–50 kDa bands
Lung dry/wet weight ratio	N.S.	N.S.
Lung tissue MMP-9 activity	N.S.	N.S
BALF PMN cell count	r: 0.8181; *p* < 0.0001	r: 0.8026; *p* < 0.0001
BALF total protein content	r: 0.6445; *p* < 0.0001	r: 0.5120; *p* < 0.001
BALF hemoglobin content	r: 0.777; *p* < 0.0001	r: 0.8673; *p* < 0.0001
Score for PMN cells in alveolar spaces (histological evaluation)	r: 0.4572; *p* < 0.05	r: 0.78; *p* < 0.0001
Score for PMN cells in the interstitium (histological evaluation)	r: 0.4572; *p* < 0.05	r: 0.785; *p* < 0.0001
Proteinaceous debris (histological evaluation)	r: 0.6153; *p* < 0.001	r: 0.65; *p* < 0.0001
Alveolar septal thickening (histological evaluation)	r: 0.4074; *p* < 0.05	r: 0.7892; *p* < 0.0001

*BALF* bronchoalveolar lavage fluid, *MMP-9* matrix metalloproteinase-9, *PMN* polymorphonuclear, *N.S.* non-significant

### Elastin degradation products in BALF of patients with exacerbated interstitial lung diseases and evidence of aspiration

**Fig. 8:**
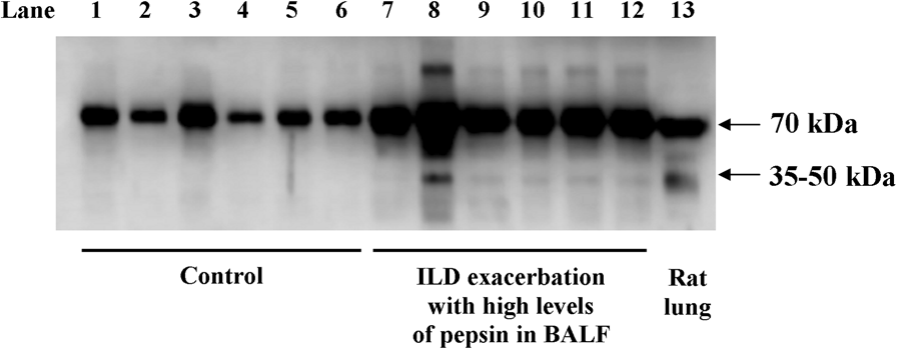
Elastin degradation products in patients with interstitial lung diseases and evidence of aspiration. Representative Western blot of soluble elastin in BALF samples obtained from patients with interstitial lung diseases and controls. Lanes 1 to 6 correspond to BALF samples from subjects without interstitial lung disease and no evidence of aspiration (negative pepsin). A single band in the 70 kDa range is detected in all samples, without evidence of smaller molecular weight elastin-derived peptides. Lanes 7 to 12 correspond to BALF samples from subjects with an exacerbation of an interstitial lung disease, with no evidence of infection and with high levels of pepsin. The 70 kDa band corresponding to tropoelastin is present in all samples, whereas the 35–50 kDa bands are detected in 5 out of 6 samples. In addition, a diffuse pattern of bands in the 50–70 kDa range is seen in all samples. Lane 13 corresponds to a BALF sample from a rat that received a single instillation of gastric fluid and was studied 12 h later (a time point at which elastin degradation products exhibit maximum levels). ILD: interstitial lung disease; h: hours

To assess the possibility of lung elastin degradation in humans with high probability of gastric contents aspiration, we studied BALF samples obtained from patients with an exacerbation of interstitial lung disease and high levels of pepsin (*n* = 6) Fig. 8 shows a representative Western blot of soluble elastin in BALF samples from these patients and from patients without interstitial lung disease and no evidence of aspiration who required bronchoscopy for the study of a lung nodule) (*n* = 6) and served as controls for this evaluation. BALF samples from subjects without interstitial lung disease and negative pepsin) (lanes 1 to 6) show a single band in the 70 kDa range, without evidence of smaller molecular weight elastin-derived peptides.

All six BALF samples from subjects with an exacerbation of an interstitial lung disease, with no evidence of infection and with high levels of pepsin (lanes 7 to 12) show the 70 kDa band seen in control samples, and a diffuse pattern of bands in the 50–70 kDa range. Five of the samples (lanes 7 to 12) show small molecular weight bands in the 35–50 kDa range as those seen in the rat BALF obtained 12 h after gastric fluid instillation (lane 13). We had access to the total cell count of these human BALF samples. Whereas in control BALFs, total cell count was homogeneously low (0.48 ± 0.2 × 10^6^ cells x ml^− 1^; range: 0.2 to 0.7), samples from exacerbated ILD patients showed variable cell counts (2,1 ± 1.3 × 10^6^ cells x ml^− 1^; range: 0.8 to 4.0). Interestingly the sample with the highest total cell count showed the greatest elastin degradation.

## Discussion

Our results show evidence of degradation of lung elastin during the early phase of ALI induced by a single instillation of gastric fluid, well before there is evidence of myofibroblasts in the alveolar structures. At this stage, there is evidence of severe derangement of the alveolar-capillary barrier and significant accumulation of PMN cells in the alveolar spaces and the interstitium, capable of expressing and discharging matrix-degrading enzymes into the extracellular space. Contrary to what we observed in the early phase (first 24 h), we did not find elastin degradation products during the phase of organization of the exudate (already evident at day 4), in which there is a marked reduction in PMN cells. These findings strongly suggest that elastin degradation occurs as a consequence of the initial injury and not as part of ECM remodeling in the context of lung tissue repair. The pattern of elastin degradation products found in our model is similar to that resulting from the effect of exogenous elastase on normal rat lung tissue in vitro*.* A similar pattern of elastin degradation was found in BALF of patients with exacerbation of an ILD without infection but with evidence of aspiration of gastric contents.

Elastin, a polymer of tropoelastin is a major component of lung ECM [21, 22]. In the normal healthy lung, tropoelastin synthesis is restricted to periods of development and growth in the perinatal life. Soluble monomers of tropoelastin are rapidly cross-linked into a network of mature insoluble elastin molecules that provides the lung elasticity, tensile strength, and stability [21]. Elastic fibers are very long-lasting and have little turnover. Although expression of the tropoelastin gene is normally absent from most adult tissues, its reactivation in severe lung injury has been recognized [33–36] consistent with the concept that mature elastin fibers that have been broken down in the lung are replaced with often excess deposition of immature elastin fibers and elastin precursors, in an unsuccessful effort to restore damaged mature elastin [23]. Alpha-SMA positive cells have been described as the site of new tropoelastin synthesis [33].

Various proteases are able to cleave elastin fibers by damaging the microfibrils and the elastin core, resulting in loss of elasticity [28, 37–39]. Markers of mature elastin degradation, mainly desmosine and isodesmosine have been commonly used and found to be present in chronic conditions such as aging, COPD [24, 40] and idiopathic pulmonary fibrosis [26]. Little is known about the role of elastic tissue destruction in acute lung injuries. There is evidence that elastin degradation can occur in association with acute lung injury characterized by fibrotic repair in experimental animals. In this regard, in the bleomycin-induced lung injury, we have described proteolytic fragmentation of the alveolar septa and enlargement of the peribronchiolar air spaces, changes that become apparent only after resolution of DAD [41]. In the same line, high levels of desmosine in BALF of animals treated with bleomycin have been documented [19, 42]. With regard to human studies, Mc Clintoch et al. [25] showed that elevated levels of urine desmosine, early in the course of ALI are associated with higher mortality rates. They also showed that ventilator-induced extracellular matrix breakdown relates to the type of ventilation used. Among patients with acute lung injury, those ventilated with less injurious ventilatory modalities had lower urine desmosine levels than those ventilated with more injurious forms of ventilation [25].

The use of antibodies to identify tropoelastin and its degradation products according to their molecular weight is very recent and most of the investigations refer to organs other than the lung [37, 43, 44]. These investigations have shown small amounts of soluble elastin with a molecular weight of 70 kDa corresponding to the tropoelastin monomer present in normal tissues [37, 43, 44] without evidence of elastin degradation. With a similar approach we were able to detect lung soluble tropoelastin (70 kDa band) in lung tissue and BALF of control animals in the present study.

After gastric fluid instillation, the density of the 70 kDa band changes with a similar pattern both in lung tissue and in BALF, with a peak at 4 h after instillation, and a progressive decline thereafter, in parallel with the progressive reduction in PMN cells. The few studies that use SDS-PAGE in other tissues in more chronic conditions, interpret the increment in the 70 kDa band density as secondary to an increment in tropoelastin synthesis [43, 44]. However, in our model it is likely that the very early increment in this protein content, that takes place well before there is evidence of myofibroblasts in the alveolar structures may represent a large initial breakdown of mature elastin, rather than proof of an early increase in tropoelastin synthesis. Myofibroblasts are considered to be the source of reactivation of tropoelastin gene expression [33] and they are seen at day 4. Thus, the very early increment in the 70 kDa band density can be seen in itself as a degradation product of mature elastin. Further support to this interpretation is provided by our finding of elastic fiber system damage when using histochemical staining.

With regard to elastin degradation products of smaller molecular weight (50 and 35 kDa), in some disease conditions not affecting the lungs, these have been interpreted as the result of tropoelastin degradation [37, 43, 44], mainly by elastolytic enzymes produced by neutrophils and macrophages. Differences in the time course of changes of both tropoelastin and small molecular weight degradation products suggest that indeed in our model, the small molecular weight degradation products result from tropoelastin degradation.

Possible mechanisms involved in elastin degradation in our model include a number of enzymes and acidic hydrolysis, among others [45–47]. However, according to our results, the acidic pH of gastric fluid is likely not responsible for elastin degradation. Instead, the destructive effects of inflammatory cells on the ECM seem to be the main factor responsible for elastin degradation. Although several enzymes are capable of elastin degradation, several pieces of evidence support a major role for neutrophil elastase in lung elastolysis in our model: a) the significant positive correlation found between elastin degradation products and the PMN cell count in BALF and in histological sections during initial ALI, b) the lack of elastin degradation products during the organization phase of the exudate, a period of time in which the number of PMN cells is significantly reduced, c) the lack of correlation between soluble elastin and lung tissue MMP-9 activity, and d) the pattern of degradation products found in our model, which is similar to that resulting from the effect of exogenous elastase on normal rat lung tissue in vitro*.* These associations will require further experiments like PMN depletion in order to confirm the role of these cells in the elastinolytic process that we have described.

Studies have revealed that elastin is not only a structural protein influencing the architecture and biomechanical properties of the ECM but also plays an active role in various physiological processes [48]. In fact, elastin-derived peptides are not only degradation products, but also bioactive moieties evoking reactions in the surrounding tissues. Thus, it has been shown that they participate in the regulation of cell adhesion, chemotaxis, migration, proliferation, protease activation, and apoptosis [48]. Although we did not study the bioactive properties of the elastin degradation products generated in our model, the molecular weight of these products is similar to that of elastase-derived peptides described as capable of inducing chemotaxis, migration, etc. in other models [49, 50]. In this sense, the elastin degradation products detected in our model are not just end products of elastin damage, but could be regarded also as important contributors to the cascade of events in the pathogenesis of lung tissue injury after gastric contents aspiration.

The present investigation provides evidence that a similar but distinct pattern of elastin degradation is present in BALF samples from patients with exacerbation of ILD with evidence of aspiration.

We speculate that in disease states in which gastroesophageal reflux is prevalent [27, 51] and possibilities of aspiration into the lung are high, the cascade of events triggered by gastric contents aspiration may be an important mechanism contributing to the elastin degradation reported in these conditions. Thus, protection of lung elastic tissue from the effects of inflammatory cell-derived proteases could be an important therapeutic target to modulate injury severity induced by gastric contents aspiration.

## Conclusions

A single instillation of gastric fluid into the rat lung induces early proteolytic degradation of elastin, in relation to the magnitude of alveolar-capillary barrier derangement. Our data suggests that PMN-derived proteases released during ALI and not the acid component of gastric fluid, are mostly responsible for this damage. Evaluation of BALF from patients with ILDs who have evidence of aspiration showed elastin degradation products similar to those found in BALF of rats treated with gastric fluid. Based on our findings and since there is consensus in that chronic damage to the elastic system of the lung can produce irreversible damage to lung architecture and loss of lung function, repeated instillations of gastric fluid in experimental animals or repeated aspiration events in humans, could contribute to long-lasting effects on lung elastic properties. We propose that protection of lung elastic tissue from the effects of inflammatory cell-derived proteases could be an important therapeutic target to modulate injury severity induced by gastric contents aspiration.

## Abbreviations

ALI: Acute lung injury
ARDS: Acute respiratory distress syndrome
BALF: Bronchoalveolar lavage fluid
COP: Cryptogenic organizing pneumonia
COPD: Chronic obstructive pulmonary diseases
DAD: Diffuse alveolar damage
ECM: Extracellular matrix
ILD: Interstitial lung diseases
IPF: Idiopathic pulmonary fibrosis
MMP-2: Matrix metalloproteinase-2
MMP-9: Matrix metalloproteinase-9
OP: Organizing pneumonia
PMN: Polymorphonuclear
SDS-PAGE: Sodium dodecyl sulphate-polyacrylamide gel electrophoresis
UIP: Usual interstitial pneumonia
α-SMA: Alpha-smooth muscle actin

## References

[1] Hu X, Lee JS, Pianosi PT, Ryu JH (2015). Aspiration-related pulmonary syndromes. Chest.

[2] Cabrera-Benitez NE, Laffey JG, Parotto M, Spieth PM, Villar J, Zhang H, Slutsky AS (2014). Mechanical ventilation-associated lung fibrosis in acute respiratory distress syndrome: a significant contributor to poor outcome. Anesthesiology.

[3] Matthay MA, Mednick G, Matthay ZA. Aspiration-induced lung injury; experimental and clinical studies. In: Vincent JL, editor. Intensive Care Medicine. New York: Springer Science and Business Media Inc; 2006. p. 359–65.

[4] Raghavendran K, Nemzek J, Napolitano LM, Knight PR (2011). Aspiration-induced lung injury. Crit Care Med.

[5] Amigoni M, Bellani G, Scanziani M, Masson S, Bertoli E, Radaelli E, Patroniti N, Di Lelio A, Pesenti A, Latini R (2008). Lung injury and recovery in a murine model of unilateral acid aspiration: functional, biochemical, and morphologic characterization. Anesthesiology.

[6] Kennedy TP, Johnson KJ, Kunkel RG, Ward PA, Knight PR, Finch JS (1989). Acute acid aspiration lung injury in the rat: biphasic pathogenesis. Anesth Analg.

[7] Knight PR, Druskovich G, Tait AR, Johnson KJ (1992). The role of neutrophils, oxidants, and proteases in the pathogenesis of acid pulmonary injury. Anesthesiology.

[8] Patel BV, Wilson MR, Takata M (2012). Resolution of acute lung injury and inflammation: a translational mouse model. Eur Respir J.

[9] Knight PR, Rutter T, Tait AR, Coleman E, Johnson K (1993). Pathogenesis of gastric particulate lung injury: a comparison and interaction with acidic pneumonitis. Anesth Analg.

[10] Teabeaut JR (1952). Aspiration of gastric contents: an experimental study. Am J Pathol.

[11] Knight PR, Davidson BA, Nader ND, Helinski JD, Marschke CJ, Russo TA, Hutson AD, Notter RH, Holm BA (2004). Progressive, severe lung injury secondary to the interaction of insults in gastric aspiration. Exp Lung Res.

[12] Raghavendran K, Davidson BA, Mullan BA, Hutson AD, Russo TA, Manderscheid PA, Woytash JA, Holm BA, Notter RH, Knight PR (2005). Acid and particulate-induced aspiration lung injury in mice: importance of MCP-1. Am J Physiol Lung Cell Mol Physiol.

[13] Mendelson CL (1946). The aspiration of stomach contents into the lungs during obstetric anesthesia. Am J Obstet Gynecol.

[14] Davidson BA, Knight PR, Wang Z, Chess PR, Holm BA, Russo TA, Hutson A, Notter RH (2005). Surfactant alterations in acute inflammatory lung injury from aspiration of acid and gastric particulates. Am J Physiol Lung Cell Mol Physiol..

[15] Appel JZ, Lee SM, Hartwig MG, Li B, Hsieh CC, Cantu E, Yoon Y, Lin SS, Parker W, Davis RD (2007). Characterization of the innate immune response to chronic aspiration in a novel rodent model. Respir Res.

[16] Downing TE, Sporn TA, Bollinger RR, Davis RD, Parker W, Lin SS (2008). Pulmonary histopathology in an experimental model of chronic aspiration is independent of acidity. Exp Biol Med (Maywood).

[17] Hartwig MG, Appel JZ, Li B, Hsieh CC, Yoon YH, Lin SS, Irish W, Parker W, Davis RD (2006). Chronic aspiration of gastric fluid accelerates pulmonary allograft dysfunction in a rat model of lung transplantation. J Thorac Cardiovasc Surg.

[18] Araos J, Ayala P, Meneses M, Contreras R, Cutiño A, Montalva R, Tazelaar H, Borzone G (2015). Resolution of lung injury after a single event of aspiration: a model of bilateral instillation of whole gastric fluid. Am J Pathol.

[19] Mecham RP. Elastin in lung development and disease pathogenesis. Matrix Biol. 2018; 10.1016/j.matbio.2018.01.005. 10.1016/j.matbio.2018.01.005 PMC6041195 29331337

[20] Liu X, Ma S, Turino G, Cantor J (2017). The pattern of elastic Fiber breakdown in Bleomycin-induced pulmonary fibrosis may reflect microarchitectural changes. Lung.

[21] Fukuda Y, Ferrans VJ (1988). Pulmonary elastic fiber degradation in paraquat toxicity. An electron microscopic immunohistochemical study. J Submicrosc Cytol Pathol.

[22] Davidson JM (1990). Biochemistry and turnover of lung interstitium. Eur Respir J.

[23] Kottmann RM, Sharp J, Owens K, Salzman P, Xiao GQ, Phipps RP, Sime PJ, Brown EB, Perry SW (2015). Second harmonic generation microscopy reveals altered collagen microstructure in usual interstitial pneumonia versus healthy lung. Respir Res.

[24] Deslee G, Woods JC, Moore CM, Liu L, Conradi SH, Milne M, Gierada DS, Pierce J, Patterson A, Lewit RA, Battaile JT, Holtzman MJ, Hogge JC, Pierce RA (2009). Elastin expression in very severe human COPD. Eur Respir J.

[25] McClintock DE, Starcher B, Eisner MD, Thompson BT, Hayden DL, Church GD, Matthay MA. Higher urine desmosine levels are associated with mortality in patients with acute lung injury, Am J Physiol Lung Cell Mol Physiol. 2006;29:566–71. 10.1152/ajplung.00457.2005 PMC2765125 16698854

[26] de Brouwer B, Drent M, van den Ouweland JMW, Wijnen PA, van Moorsel CHM, Bekers O, Grutters JC, White ES, Janssen R (2018). Increased circulating desmosine and age-dependent elastinolysis in idiopathic pulmonary fibrosis. Respir Res.

[27] Ghebre YT, Raghu G (2016). Idiopathic pulmonary fibrosis: novel concepts of proton pump inhibitors as Antifibrotic drugs. Am J Respir Crit Care Med.

[28] Blázquez-Prieto J, López-Alonso I, Huidobro C, Albaiceta GM. The emerging role of neutrophils in repair after acute lung injury. Am J Respir Cell Mol Biol. 2018; 10.1165/rcmb.2018-0101PS. 10.1165/rcmb.2018-0101PS 29708395

[29] Matute-Bello G, Downey G, Moore BB, Groshong SD, Matthay MA, Slutsky AS, Kuebler WM (2011). An official American Thoracic Society workshop report: features and measurements of experimental acute lung injury in animals. Am J Respir Cell Mol Biol.

[30] Lillie RD, Fullmer HM (1976). Histopathological technic and practical histochemistry.

[31] Toth M, Sohail A, Fridman R (2012). Assessment of gelatinases (MMP-2 and MMP-9) by gelatin zymography. Methods Mol Biol.

[32] Sokal RR, Rohlf FJ (1981). Biometry.

[33] Mariani TJ, Crouch E, Roby JD, Starcher B, Pierce RA (1995). Increased elastin production in experimental granulomatous lung disease. Am J Pathol.

[34] Starcher BC, Kuhn C, Overton JE (1978). Increased elastin and collagen content in the lungs of hamsters receiving an intratracheal injection of bleomycin. Am Rev Respir Dis.

[35] Raghow R, Lurie S, Seyer JM, Kang AH (1985). Profiles of steady state levels of messenger RNAs coding for type I procollagen, elastin, and fibronectin in hamster lungs undergoing bleomycin-induced interstitial pulmonary fibrosis. J Clin Invest.

[36] Rocco PR, Negri EM, Kurtz PM, Vasconcellos FP, Silva GH, Capelozzi VL, Romero PV, Zin WA (2001). Lung tissue mechanics and extracellular matrix remodeling in acute lung injury. Am J Respir Crit Care Med.

[37] Wei PC, Tsai CH, Chiu PS, Lai SC (2011). Matrix metalloproteinase-12 leads to elastin degradation in BALB/c mice with eosinophilic meningitis caused by Angiostrongylus cantonensis. Int J Parasitol.

[38] Campbell EJ, Senior RM, Welgus HG (1987). Extracellular matrix injury during lung inflammation. Chest.

[39] Heinz A, Jung MC, Duca L, Sippl W, Taddese S, Ihling C, Rusciani A, Jahreis G, Weiss AS, Neubert RH, Schmelzer CE (2010). Degradation of tropoelastin by matrix metalloproteinases -cleavage site specificities and release of matrikines. FEBS J.

[40] He J, Turino GM, Lin YY (2010). Characterization of peptide fragments from lung elastin degradation in chronic obstructive pulmonary disease. Exp Lung Res.

[41] Borzone G, Moreno R, Urrea R, Meneses M, Oyarzún M, Lisboa C (2001). Bleomycin-induced chronic lung damage does not resemble human idiopathic pulmonary fibrosis. Am J Respir Crit Care Med.

[42] Idell S, Thrall RS, Maunder R, Martin TR, McLarty J, Scott M, Starcher BC (1989). Bronchoalveolar lavage desmosine in bleomycin-induced lung injury in marmosets and patients with adult respiratory distress syndrome. Exp Lung Res.

[43] Chou PH, Lai SC (2011). Elevated concentrations of matrix metalloproteinase-12 and elastin degradation products in the sera of pregnant women infected with toxoplasma gondii. Ann Trop Med Parasitol.

[44] Akima T, Nakanishi K, Suzuki K, Katayama M, Ohsuzu F, Kawai T (2009). Soluble elastin decreases in the progress of atheroma formation in human aorta. Circ J.

[45] Collins JF, Fine R (1981). The enzymatic digestion of elastin at acidic pH. Biochim Biophys Acta.

[46] Umeda H, Nakamura F, Suyama K (2001). Oxodesmosine and isooxodesmosine, candidates of oxidative metabolic intermediates of pyridinium cross-links in elastin. Arch Biochem Biophys.

[47] Hayashi A, Ryu A, Suzuki T, Kawada A, Tajima SH (1998). In vitro degradation of tropoelastin by reactive oxygen species. Arch Dermatol Res.

[48] Gaggar A, Weathington N (2016). Bioactive extracellular matrix fragments in lung health and disease. J Clin Invest.

[49] Senior RM, Griffin GL, Mecham RP (1980). Chemotactic activity of elastin-derived peptides. J Clin Invest.

[50] Hunninghake GW, Davidson JM, Rennard S, Szapiel S, Gadek JE, Crystal RG (1981). Elastin fragments attract macrophage precursors to diseased sites in pulmonary emphysema. Science.

[51] Cardasis JJ, MacMahon H, Husain AN. The spectrum of lung disease due to chronic occult aspiration. Ann Am Thorac Soc. 2014;11:865–73. 10.1513/AnnalsATS.201310-360OC 24950025

